# Use of Transcutaneous Electrical Nerve Stimulation (TENS) for Pain Management During Intrauterine Device Insertion: A Case Series

**DOI:** 10.7759/cureus.69324

**Published:** 2024-09-13

**Authors:** Merlin Perez Navarro, Benny Esquenazi

**Affiliations:** 1 Obstetrics and Gynecology, Herbert Wertheim College of Medicine, Florida International University, Miami, USA

**Keywords:** contraception, iud insertion, pain management, tens unit, transcutaneous electrical nerve stimulation

## Abstract

This case series details the use of transcutaneous electrical nerve stimulation (TENS) for pain management during intrauterine device (IUD) insertion in three different patients. We used a 100 mm horizontal line visual analog scale (VAS) to assess pain associated with the procedure. Case 1 is a 39-year-old primiparous female with a past medical history of depression who presented to the clinic for Mirena IUD insertion. The patient rated overall pain during the procedure as 10 mm, with the most discomfort during IUD placement inside the uterus, which she rated 20 mm. Case 2 is a 16-year-old nulliparous female with no significant past medical history who presented for Mirena IUD insertion. The patient rated overall pain during the procedure as 40 mm, with the most discomfort during IUD placement inside the uterus, which she rated 45 mm. Case 3 is a 37-year-old multiparous female with no significant past medical history who also received a Mirena IUD. She rated overall pain during the procedure as 20 mm. All patients reported lower pain scores when compared to previously reported median pain scores associated with the procedure, highlighting the analgesic effects of TENS.

## Introduction

Transcutaneous electrical nerve stimulation (TENS) is a cost-effective device used to manage both acute and chronic pain conditions. It works by delivering alternating current through cutaneous electrodes placed near the pain site. The effectiveness of TENS can be adjusted by modifying the pulse frequency and intensity settings [1]. It provides analgesia through a complex neuronal network involving both central and peripheral mechanisms [1]. TENS activates descending inhibitory pathways and mediates increased neuronal activity, specifically in the rostral ventromedial medulla (RVM), periaqueductal gray (PAG), and spinal cord. High-frequency (HF) TENS provides pain relief by stimulating endogenous inhibitory mechanisms in the central nervous system, which involve gamma-aminobutyric acid (GABA), opioid, and muscarinic receptors [1]. Low-frequency (LF) TENS, on the other hand, utilizes descending inhibitory pathways, particularly the PAG-RVM pathway, to activate GABA, opioid, muscarinic, and serotonin receptors. This process reduces the activity of dorsal horn neurons and alleviates pain [2,3]. Both applications provide adequate analgesia for certain acute and chronic pain conditions, specifically when applied at a strong, nonpainful intensity. When used for intrauterine device (IUD) insertion, TENS electrodes stimulate the nerve roots at the T10-L1 and S2-S4 dermatomal levels, providing analgesia to the whole uterus.

## Case presentation

Case 1

A 39-year-old female, with a past medical history of depression, presents to the clinic for IUD insertion. Menarche occurred at age 13; menstrual cycles are regular, occur every 29 days, and last five days. She is sexually active with her husband and uses condoms for contraception. Her last menstrual period was approximately 25 days ago. Her last pap smear, done two years ago, was normal. She has no history of sexually transmitted diseases (STDs). Obstetric history includes a term vaginal delivery of a baby boy seven years ago, without complications. The patient had a Mirena IUD for six years, which was removed three months ago, and now presents for reinsertion of a new device. She reported moderate discomfort during the previous insertion. The patient was informed about the purpose of TENS and subsequently consented to its use during the procedure. The electrodes were positioned parallel to the spinal cord at the T10-L1 and S2-S4 levels to stimulate the nerve roots connected to the uterus. The patient was instructed to adjust the TENS intensity to the highest level that did not cause pain, as higher intensities are linked to improved analgesia. For this patient, the intensity ranged from 27 to 28 mA, and the HF TENS varied randomly between 80 and 100 Hz. We chose the random frequency method as evidence suggests that it provides better pain control compared with conventional fixed frequencies [4]. The pulse duration was set at 350 microseconds since previous studies have indicated that a pulse duration above 250 microseconds produces better pain relief [4]. The patient was then placed in the lithotomy position, and her vagina and cervix were cleaned with Betadine. The cervix was grasped with a single-tooth tenaculum, and the Mirena IUD was inserted without difficulty. The strings were trimmed to 3 cm, with no complications. A transabdominal ultrasound confirmed that the IUD was in the fundus of the uterus in the usual position. A 100 mm horizontal visual analog scale (VAS) was used to assess pain associated with the procedure; 0 mm indicated no pain, and 100 mm indicated the worst possible pain. The patient rated her overall pain during the procedure as 10 mm, with the most discomfort during actual IUD placement inside the uterus, which she rated as 20 mm. She did not experience any adverse effects, such as pain or rash, at the electrode site. Overall, she was satisfied with the procedure and experienced minimal pain.

Case 2

A 16-year-old female, with no significant past medical history, presents to the clinic for IUD insertion. Menarche occurred at age 12; menstrual cycles are regular, occur every 28-30 days, and last five to seven days. She is sexually active with her boyfriend and does not use contraception. Her last menstrual period was 28 days ago. She has no history of STDs or previous pregnancies. Consent was obtained for the use of TENS during the procedure. Electrodes were placed parallel to the spinal cord at the T10-L1 and S2-S4 levels. She was instructed to increase the TENS intensity to the maximum nonpainful level, ranging from 28 to 30 mA. HF TENS varied randomly between 80 and 100 Hz. The pulse duration was set at 350 microseconds. The patient was placed in the lithotomy position; Betadine was used to clean the vagina and cervix, and the Mirena IUD was inserted without difficulty. The strings were trimmed to 3 cm, with no complications. A transabdominal ultrasound confirmed proper IUD placement in the uterus. The patient rated her overall pain during the procedure as 40 mm, with the most discomfort during the actual IUD placement inside the uterus, which she rated as 45 mm. She did not experience any adverse effects, such as pain or rash, at the electrode site. The patient was satisfied with the procedure.

Case 3

A 37-year-old female, with no significant past medical history, presents to the clinic for IUD insertion. Menarche occurred at age 15; menstrual cycles are regular, occurring every 31 days and lasting four to five days. She is sexually active with her husband and uses condoms for contraception. Her last menstrual period was approximately 28 days ago. Her last pap smear, done two years ago, was normal. She has no history of STDs. Obstetric history includes three C-sections: a primary cesarean of a term baby boy in 2016, a repeat cesarean of a term baby boy in 2019, and a repeat cesarean of a term baby boy in 2021. Consent was obtained for the use of TENS during the procedure. The electrodes were positioned parallel to the spinal cord at the T10-L1 and S2-S4 levels. For this patient, the intensity ranged from 28 to 29 mA. HF TENS varied randomly between 80 and 100 Hz. The pulse duration was set at 350 microseconds. The patient was placed in the lithotomy position; Betadine was used to clean the vagina and cervix, and the Mirena IUD was inserted without difficulty. The strings were trimmed to 3 cm, with no complications. A transabdominal ultrasound confirmed proper IUD placement in the uterus. The patient rated her overall pain during the procedure as 20 mm. She did not experience any adverse effects, such as pain or rash, at the electrode site. The patient was satisfied with the procedure.

## Discussion

IUDs are one of the most reliable and commonly used methods of contraception. Two types of IUDs are currently used in the United States: one that contains copper and one that contains levonorgestrel. They are both similarly efficient in pregnancy prevention, with failure rates of 0.08% for the copper IUD and 0.02% for the hormonal IUD [5]. The devices are a type of long-acting reversible contraception (LARC) that has become increasingly popular since the 1990s [6]. There are four types of hormonal IUDs available in the United States: Mirena, Liletta, Kyleena, and Skyla. They all contain the same hormone, levonorgestrel, but in different quantities: 13.5 mg (Skyla), 19.5 mg (Kyleena), and 52 mg (Liletta, Mirena). They also provide different lengths of protection, ranging from three to eight years of coverage. They are all shaped like a T, with the horizontal part of the T positioned along the top of the endometrial cavity [5]. Their mechanism of action involves cessation of ovulation and thickening of cervical mucus to inhibit the motility of sperm [7]. Indications for use include contraception, and the 52 mg device is additionally approved for managing menorrhagia and for protective effects on the endometrium during hormone replacement therapy [8]. Contraindications for the use of hormonal IUDs include pregnancy or suspected pregnancy, active sexually transmitted infections, a distorted uterine cavity, abnormal uterine bleeding, reproductive tract cancers, current or recent history of breast cancer, liver tumors, and acute liver disease [5]. IUDs offer numerous benefits, such as their effectiveness, reversibility, ease of use, and high levels of patient satisfaction. Despite their benefits, many women choose alternative methods of contraception due to fear of pain associated with the insertion procedure. Cervical pain is mediated by S2-S4 parasympathetic nerves and the T10-L1 sympathetic fibers that innervate the uterine fundus. Although the level of pain varies among women, most experience mild to moderate discomfort during the procedure. Occasionally, pain can be severe and associated with nausea and weakness. It is also common for some pain to persist for a few days after insertion [9]. Several factors may predict increased pain during the procedure, including nulliparity, history of dysmenorrhea, age greater than 30 years, not currently breastfeeding, and a longer interval since the last pregnancy or menses [9]. A cohort study published in 2015 revealed that women who had not had a previous vaginal delivery experienced significantly higher pain during IUD insertion compared with women who had had a previous vaginal delivery, with median scores of 6 and 3 (on a pain scale of 1-10), respectively [10]. Additionally, women who had a previous vaginal delivery experienced significantly lower actual pain than expected [10]. For many years, there has been a continued search for better pain management during IUD insertion. Available methods for pain management include oral pain medications such as prophylactic nonsteroidal anti-inflammatory drugs (NSAIDs) and tramadol, misoprostol, nitroprusside, and paracervical nerve blocks [9]. Among all the options, prophylactic NSAIDs and paracervical nerve blocks are the most commonly used and also the most efficient, although many studies suggest these methods do not significantly reduce pain [9].

This case series explores the use of TENS devices to reduce pain during IUD insertion. The device has shown efficacy in treating acute and chronic pain conditions such as postoperative pain, lumbar pain, osteoarthritis pain, fibromyalgia, and neuropathic pain [1]. It delivers alternating current via cutaneous electrodes and provides analgesia through complex central and peripheral mechanisms. HF TENS has been found to boost the levels of β-endorphins in both the cerebrospinal fluid and bloodstream. It produces pain relief by stimulating opioid receptors in the RVM and influencing synaptic transmission in the ventrolateral periaqueductal gray (PAG) [11]. Additionally, HF TENS enhances muscarinic receptors (M1 and M3) and GABA-A receptors in the spinal cord [3]. Similarly, LF TENS also activates μ-opioid receptors in the spinal cord and affects synaptic transmission in the ventrolateral PAG [11]. Beyond this, LF TENS improves muscarinic M1 and M3 and GABA-A and receptor activity in the spinal cord and also stimulates serotonin 5-HT2A and 5-HT3 receptors, leading to an increased release of serotonin [12]. Ultimately, both HF and LF TENS use descending inhibitory pathways to reduce dorsal horn neuronal activity and decrease pain. Previous studies have demonstrated that HF TENS causes a reduced release of the excitatory neurotransmitters glutamate and substance P in the spinal cord dorsal horn of animals with inflammation, blunting excitation, and neuronal sensitization [13,14]. Another mechanism by which TENS provides analgesia is by activation of peripheral α-2a-adrenergic receptors [15].

The use of TENS during IUD insertion has not yet been explored. However, its use in similar gynecological procedures, including endometrial biopsy and hysteroscopy, has been reported. A randomized controlled trial demonstrated that TENS causes a statistically significant reduction in pain and increases patient satisfaction during office hysteroscopy [4]. Another randomized controlled trial showed that TENS was successful in significantly reducing pain 15 minutes post-endometrial biopsy [16].

## Conclusions

IUDs are one of the most reliable and commonly used methods of contraception in the United States. They offer many benefits, including efficacy, reversibility, ease of use, and patient satisfaction. Despite these advantages, many women are hesitant to get an IUD due to concerns about pain during the procedure. Most women experience mild-to-moderate discomfort, although some report severe pain. Effective pain management is therefore important, but current methods are not always satisfactory, nor are they widely used by gynecologists. This may be due to a lack of guidelines and research studies on the topic. TENS is a small device commonly used for certain acute and chronic pain conditions. Although not yet studied for its use in IUD insertion, it has shown promising analgesic properties in similar gynecological procedures. Previous studies suggest that, for optimal pain management, TENS intensity should be set to the maximum nonpainful level, along with HF TENS that randomly varies between 80 and 100 Hz, and duration of pulse above 250 microseconds. This case series displays a positive analgesic response to TENS during IUD insertion in three different patients. Pain scores in our patients can be compared to previously reported median pain scores of 6 (corresponding to 60 mm on the VAS) for women with no previous vaginal delivery and 3 (corresponding to 30 mm on the VAS) for those with a previous vaginal delivery. It also highlights that nulliparity is a risk factor for increased pain during the procedure, as the highest pain score was reported by our nulliparous patient. To our knowledge, a few clinical trials are underway to study the use of TENS for IUD insertion pain control. With the publication of this case series, we aim to inform the scientific community about an effective pain management method for a commonly performed and often uncomfortable gynecological procedure: IUD insertion.
